# A Plasmodesmal Glycosyltransferase-Like Protein

**DOI:** 10.1371/journal.pone.0058025

**Published:** 2013-02-26

**Authors:** Lisa Zalepa-King, Vitaly Citovsky

**Affiliations:** Department of Biochemistry and Cell Biology, State University of New York, Stony Brook, New York, United States of America; Iowa State University, United States of America

## Abstract

Plasmodesmata (Pd) are plant intercellular connections that represent cytoplasmic conduits for a wide spectrum of cellular transport cargoes, from ions to house-keeping proteins to transcription factors and RNA silencing signals; furthermore, Pd are also utilized by most plant viruses for their spread between host cells. Despite this central role of Pd in the plant life cycle, their structural and functional composition remains poorly characterized. In this study, we used a known Pd-associated calreticulin protein AtCRT1 as bait to isolate other Pd associated proteins in *Arabidopsis thaliana*. These experiments identified a beta-1,6-N-acetylglucosaminyl transferase-like enzyme (AtGnTL). Subcellular localization studies using confocal microscopy observed AtGnTL at Pd within living plant cells and demonstrated colocalization with a Pd callose-binding protein (AtPDCB1). That AtGnTL is resident in Pd was consistent with its localization within the plant cell wall following plasmolysis. Initial characterization of an Arabidopsis T-DNA insertional mutant in the *AtGnTL* gene revealed defects in seed germination and delayed plant growth.

## Introduction

Perhaps one of the most intriguing, yet least studied, aspects of intercellular communication and transport in most higher eukaryotes, from mammals to plants, is traffic of macromolecular complexes through direct cytoplasmic bridges between the adjacent cells. These cytoplasmic intercellular connections, termed tunneling nanotubes (TNTs) [1], [2] in mammals and plasmodesmata (Pd) [3]–[5] in plants, are involved in such major cellular events as transfer of organelles and membrane-bound vesicles between mammalian cells [1] and spread of regulatory molecules, such as different transcription factors and RNA silencing signals [6]–[10], between plant cells. Furthermore, these transport mechanisms are subverted by pathogens for their movement between the host cells [2], [11]. Thus, the importance of Pd in plant physiology, development, morphogenesis, and interactions with biotic and abiotic environmental factors is impossible to overestimate, yet our understanding of their molecular composition is still incomplete.

Pd are lined with the plasma membrane, and their central region is occupied by the ER, which spans Pd and forms a continuum between the adjacent cells [4], [12]. The space between the ER and the plasma membrane contains permeable channels [13], through which molecules move from cell to cell [14]. This space, as well the intra-Pd plasma membrane and trans-Pd ER, contain numerous proteins, the identity of which has begun unravel only in the course of the last two decades. To date, Pd have been shown to contain or associate with calreticulin [15]–[18], a beta-1,3-glucanase [19], type I membrane receptor-like proteins (PDLPs) [20], a protein kinase [21], Pd callose binding proteins (PDCBs) [22], class 1 reversibly glycosylated polypeptides (^C1^RGPs) [23], and actin/myosin filaments [24], [25]. The hunt for additional Pd components continues, and this communication reports the discovery of a Pd-associated core 2/I branching beta-1,6-N-acetylglucosaminyl transferase-like protein (GnTL).

## Results

### Identification of AtGnTL

**Figure 1:**
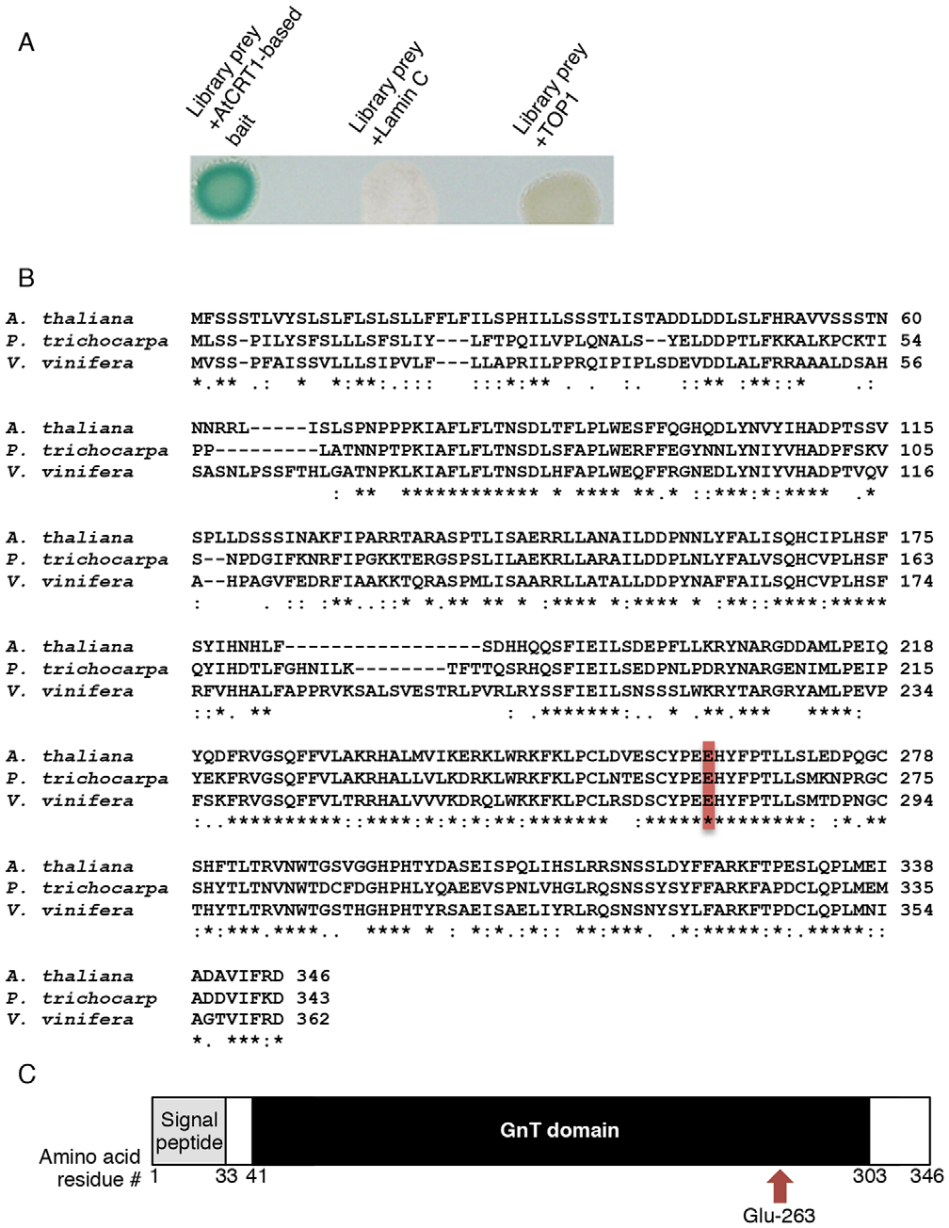
AtGnTL belongs to the acetylglucosaminyl transferase family. (A) Identification of an AtCRT1 interactor in the Y2H library screen. Cells expressing the indicated combinations of proteins were grown on leucine/tryptophan-deficient medium and analyzed for ß-galactosidase activity. (B) Amino acid sequence alignment of the *A. thaliana* GnTL (CAA05894) with its homologs from *P. trichocarpa* and grape (*V. vinifera*). Symbol designations: “*” identical residues, “:” conserved substitutions, “.” semi-conserved substitutions. The conserved glutamic acid residue likely involved in the enzymatic activity is indicated by box, and gaps introduced for alignment are indicated by dashes. Alignment was performed using the ClustalW algorithm (http://www.genebee.msu.su/clustal/advanced.html). (C) Schematic representation of the AtGnTL protein domains. Positions of amino acid residues delimiting each of the indicated domains are shown.

To define better the complement of the Pd-associated proteins, we searched our yeast two-hybrid (Y2H) cDNA library from *Arabidopsis thaliana* [26], [27] for interactors with Arabidopsis calreticulin, AtCRT1, known to accumulate within Pd in several plant species, including Arabidopsis. To avoid non-specific interactions via calcium ion-binding domains of calreticulin, we used as bait a fragment of AtCRT1 that lacked these sequences. These experiments isolated a cDNA prey encoding a protein product that interacted with the AtCRT1-based bait (Fig. 1A). Amino acid sequence analysis of this interactor protein, broadly designated as a putative glucosaminyl transferase-like enzyme (AtGnTL, AGI code At3g52060, GenBank accession number NM_180350), revealed that it belongs to the annotated family of core 2/I branching beta-1,6-N-acetylglucosaminyl transferases, with members in more than 19 plant species, including such agronomically important and diverse crops as poplar *(Poulus trichocarpa,* XP_002315417) and grape (*Vitis vinifera*, XP_00226454) (Fig. 1A). Fig. 1B shows that AtGnTL, a 346-residue protein, contains two distinct functional domains, an amino-terminal signal peptide, inherent to endoplasmic eukaryotic proteins [28], [29], which is followed by a catalytic domain (GnT) of a Branch family/glycosyltransferase family 14 (Fig. 1B). This latter domain is found in two different beta-1,6-N-acetylglucosaminyltransferase enzymes, I-branching enzyme and core-2 branching enzyme (Pfam/Interpro database entries 02485/IPR003406), that catalyze the transfer of a specific activated sugar moiety from a donor molecule to an acceptor via a glycosidic bond. Further protein domain analysis using the Pfam database (http://pfam.sanger.ac.uk/) demonstrated that AtGnTL contains a conserved glutamic acid residue (Glu-263 in AtGnTL) potentially important for the enzymatic activity (Fig. 1).

**Figure 2:**
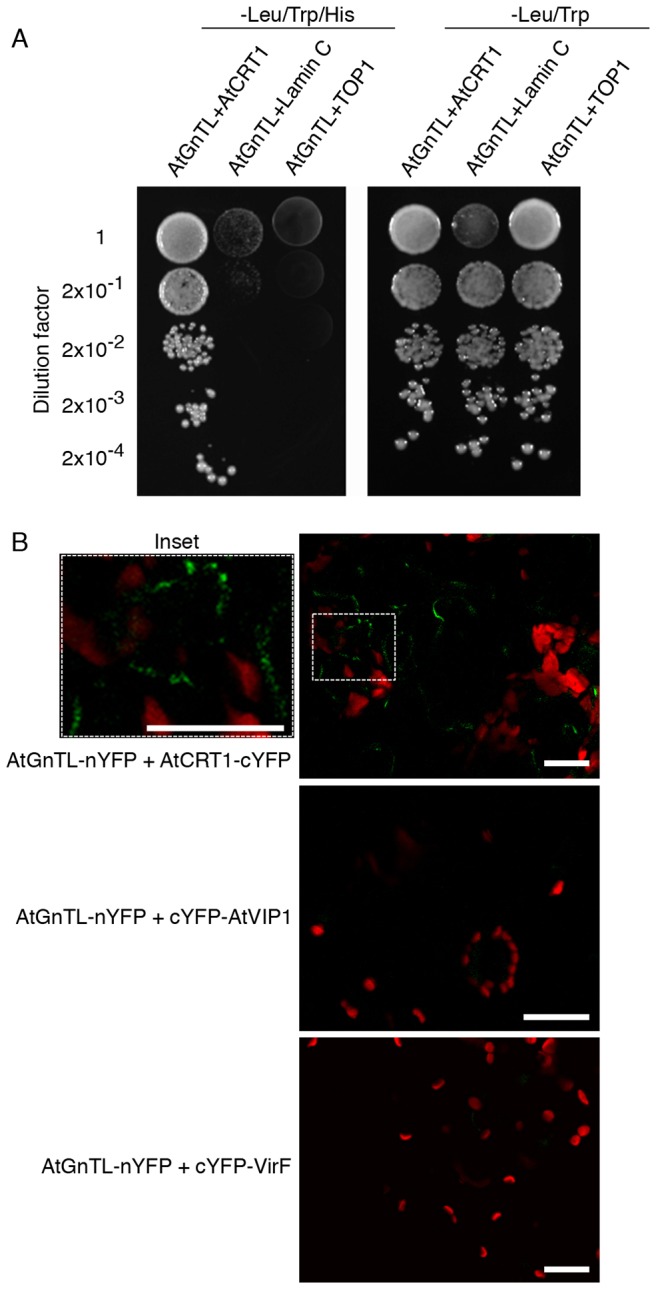
AtGnTL interacts with AtCRT1. (A) Interaction in the Y2H system. The indicated dilutions of cell cultures were grown on leucine/tryptophan-deficient medium either in the absence (left panel) or in the presence of histidine (right panel); in this assay system, cell growth without histidine represents the selective condition for protein-protein interaction. (B) Interaction in the BiFC assay in *N. benthamiana*. Inset: magnified image of the BiFC signal within the are indicated by dashed rectangle. YFP fluorescence is in green, plastid autofluorescence is in red. Images are single confocal sections. Bars = 20 µm.

The interaction between AtGnTL and full-length AtCRT1 was demonstrated in yeast and in plant cells. Fig. 2A shows that AtGnTL interacted with AtCRT1 in the Y2H system, and that this interaction was specific because it did not occur with topoisomerase 1 (TOP1) or with lamin C, known non-specific Y2H activators best suited to eliminate false positive interactions [30], [31]. Specifically, co-expression of AtGnTL with AtCRT1, but not with topoisomerase I or lamin C, activated the *HIS3* reporter gene (Fig. 2A, left panel). Under the non-selective conditions, all combinations of the tested proteins resulted in the efficient cell growth (Fig. 2A, right panel).

The Y2H data were then confirmed by an independent assay using bimolecular fluorescence complementation (BiFC), in which protein interaction is monitored in vivo, directly within living plant cells; furthermore, this approach simultaneously determines subcellular localization of the interacting proteins [32]. The AtGnTL-AtCRT1 interaction was tested in *Nicotiana benthamiana*, a choice plant for transient gene expression experiments. Fig. 2B shows that nYFP-tagged AtGnTL interacted with AtCRT1-cYFP in planta, resulting in the YFP fluorescence. This recognition of AtCRT1was specific because it was not observed with cYFP-tagged unrelated Arabidopsis VIP1 and Agrobacterium VirF proteins (Fig. 2B). Importantly, most of the population of the interacting AtGnTL and AtCRT1 proteins accumulated at distinct punctate locations at the cell periphery (Fig. 2B, inset), which are diagnostic of plasmodesmata (Pd) [,17,18,20,33]–[38], whereas some of the signal localized in cytoplasmic microbodies, possibly aggregates (Fig. 2B).

### AtGnTL localizes to Pd

**Figure 3:**
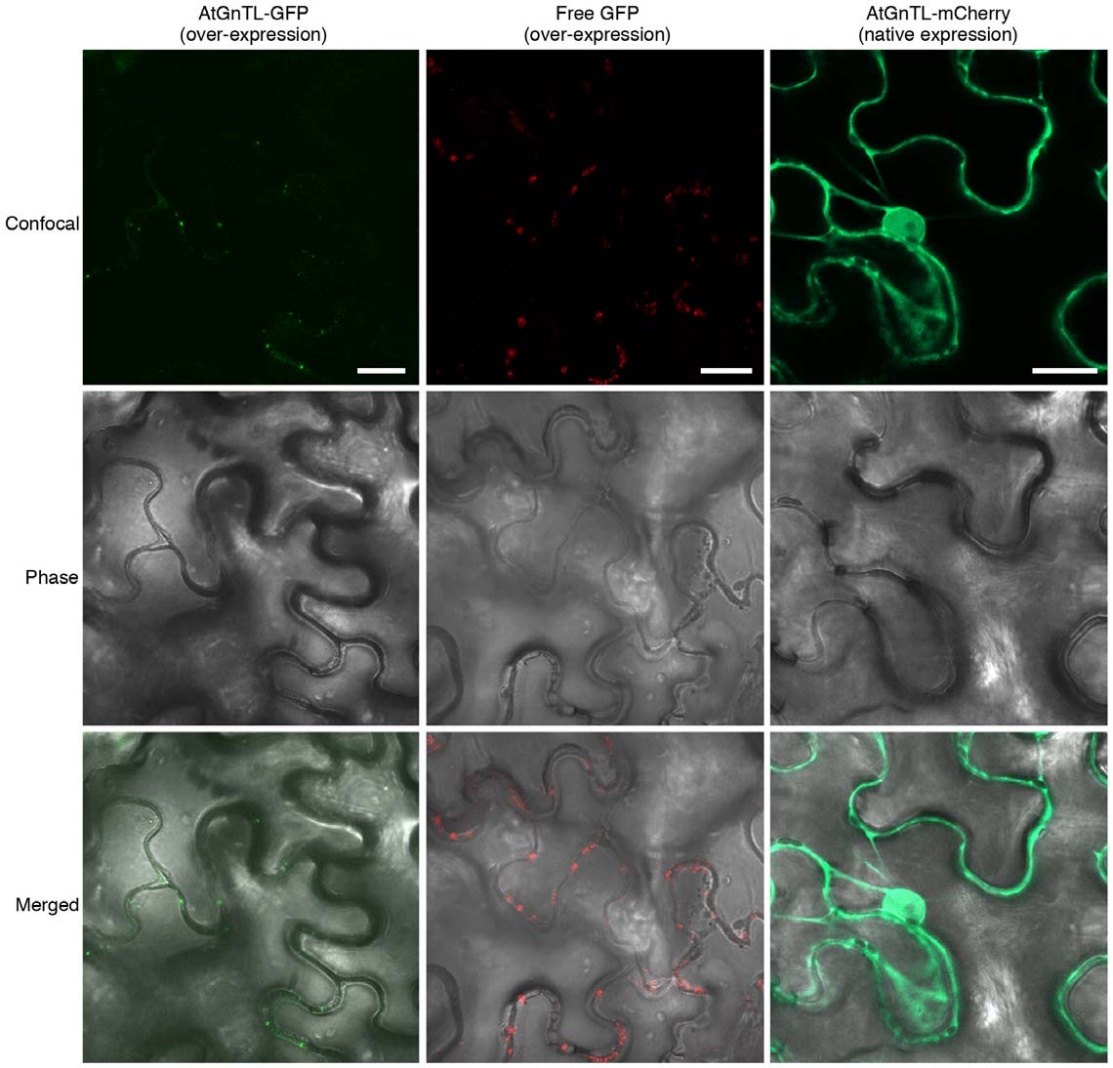
Pd-like punctate localization of AtGnTL in *N. benthamiana*. GFP fluorescence is in green, mCherry fluorescence is in red. Images are single confocal sections. Bars = 20 µm.

The notion that AtGnTL is a plasmodesmal protein was examined in further detail. Fig. 3 shows that GFP- or mCherry-tagged AtGnTL expressed in *N. benthamiana* leaf epidermis either from a constitutive 35S CaMV promoter or from its native promoter, respectively, accumulated in the Pd-like puncta. This punctate accumulation pattern was not affected by the nature of the protein tag, i.e., GFP or mCherry, and it was not observed with a free tag (Fig. 3).

**Figure 4:**
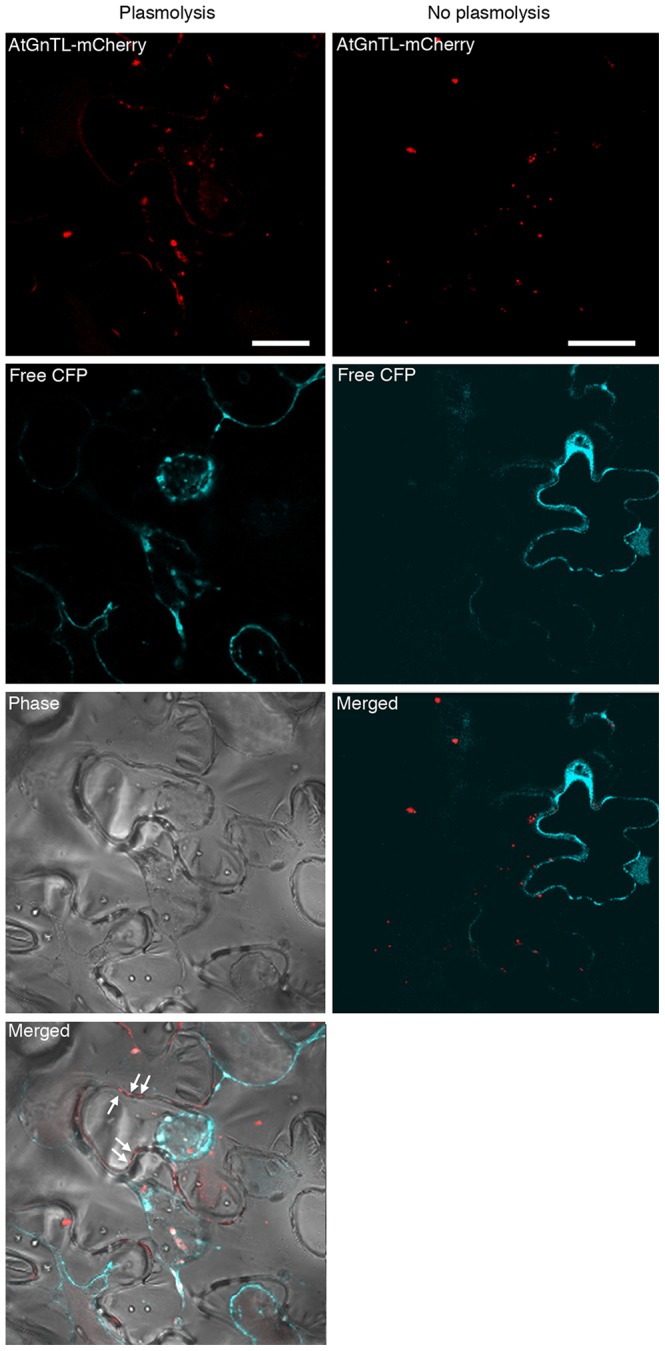
Subcellular localization of AtGnTL-mCherry in plasmolysed and non-plasmolysed tissues of *N. benthamiana*. mCherry fluorescence is in red, CFP fluorescence is in blue; plastid autofluorescence was filtered out. Punctate mCherry fluorescence pattern at the periphery of the plasmolysed cells visible in the merged phase/fluorescence images is indicated by arrows. Note that, because, without plasmolysis, the CFP signal outlines the cells, we did not include the phase images of non-plasmolysed cells. Images are single confocal sections. Bar = 20 µm.

Another characteristic feature of Pd is that they are embedded within the cell wall. We demonstrated that the AtGnTL-containing puncta indeed reside in the cell wall using the plasmolysis assay. During plasmolysis, only cell wall components retain their original localization pattern, whereas the cell cytoplasm and the plasma membrane become displaced and relocated following physical shrinking of the plasmolysed cells [39]. Fig. 4 shows that, following plasmolysis, the cell cytoplasm, visualized by transiently-expressed free CFP, indeed detached from the cell wall, with the cell content becoming compressed in the center of the cell interior. This detachment was best visible when the fluorescence data were superimposed over the phase images of the whole cells. In contrast, AtGnTL-mCherry coexpressed in the same cell retained its punctate localization pattern at the cell periphery (Fig. 4, arrows), indicating that these puncta are situated within the cell wall; note however, that some of the AtGnTL-mCherry population remained associated with the cytoplasm, apparently in microaggregates. As expected (see Fig. 3), AtGnTL-mCherry accumulated in the Pd-like puncta in non-plasmolysed cells (Fig. 4).

**Figure 5:**
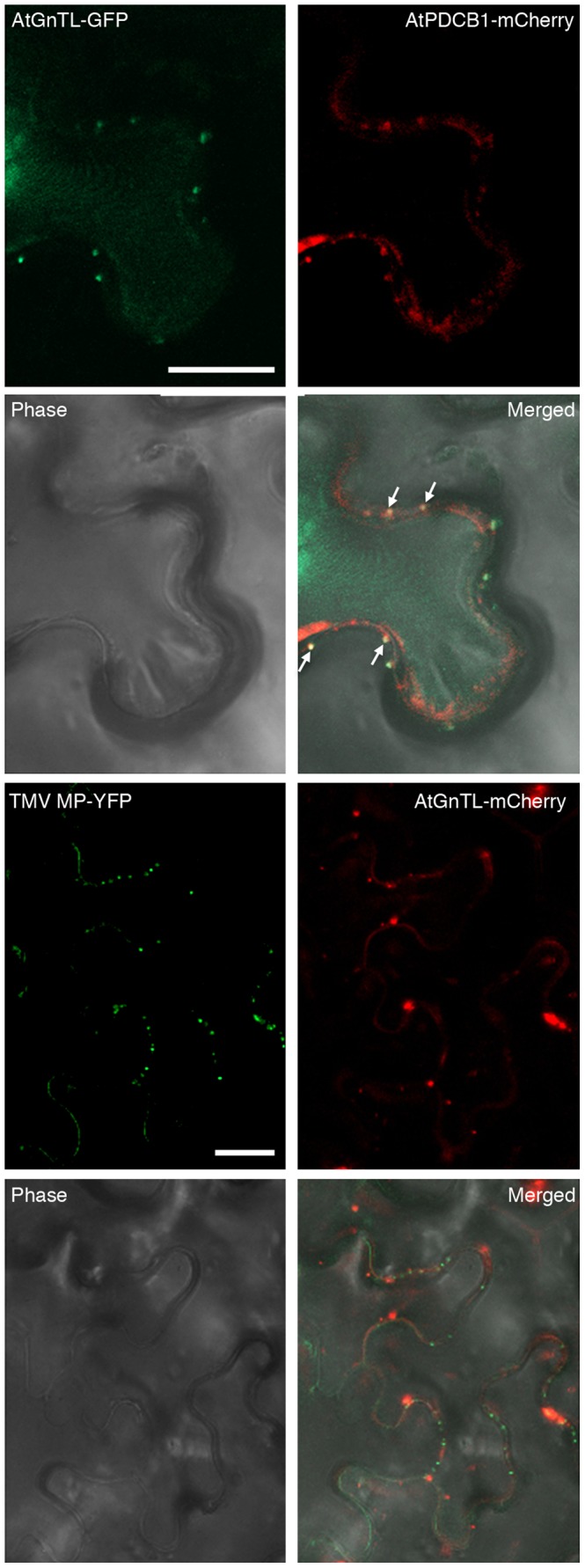
Colocalization of AtGnTL-GFP with AtPDCB1-mCherry, but not with TMV MP-mCherry, in *N. benthamiana*. GFP or YFP fluorescence is in green, mCherry fluorescence is in red, overlapping GFP/mCherry fluorescence is in yellow and is indicated by arrows; plastid autofluorescence was filtered out. Images are single confocal sections. Bars = 20 µm.

Finally, we examined whether AtGnTL actually colocalizes with a Pd marker, a cellular protein known to reside within Pd. To this end, we selected an Arabidopsis GPI-anchor callose binding protein AtPDCB1 which specifically localizes to the neck region of Pd [22], [40]. We transiently coexpressed in *N. benthamiana* leaves AtGnTL tagged with GFP and AtPDCB1 tagged with mCherry, and analyzed the distribution of the corresponding fluorescent signals. Fig. 5 shows that both proteins accumulated in the characteristic punctate patterns on the cell periphery, and that many, but not all, of such puncta overlapped each other (arrows), indicating colocalization. Note that, consistent with previous data [22], [40], AtPDCB1 also exhibited some cell wall-associated signal between puncta; obviously, these areas represented the non-overlapping signal (Fig 5). Quantification of colocalization based on the number of individual signal puncta formed by each protein and on those of them that colocalized, indicated that 80% of AtGnTL-GFP colocalized with AtPDCB1-mCherry, and 49% of AtPDCB1-mCherry colocalized with AtGnTL-GFP.

Interestingly, AtGnTL practically did not colocalize with the *Tobacco mosaic virus* (TMV) cell-to-cell movement protein (MP) (Fig. 5), which also resides in Pd, forming distinct punctate patterns [41], but accumulates in the Pd inner areas, often within the central cavity [42]. Quantification of these data indicated only 6% of AtGnTL-mCherry/TMV MP-YFP colocalization and 3% of TMV MP-YFP/AtGnTL-mCherry colocalization. These observations suggest that AtGnTL and TMV MP may target different regions of Pd; this is unlike AtCRT which most likely accesses both Pd locations as it interacts with AtGnTL as well as with TMV MP [17]. These findings raise an interesting possibility of differential Pd targeting and/or localization patterns for different proteins.

**Figure 6:**
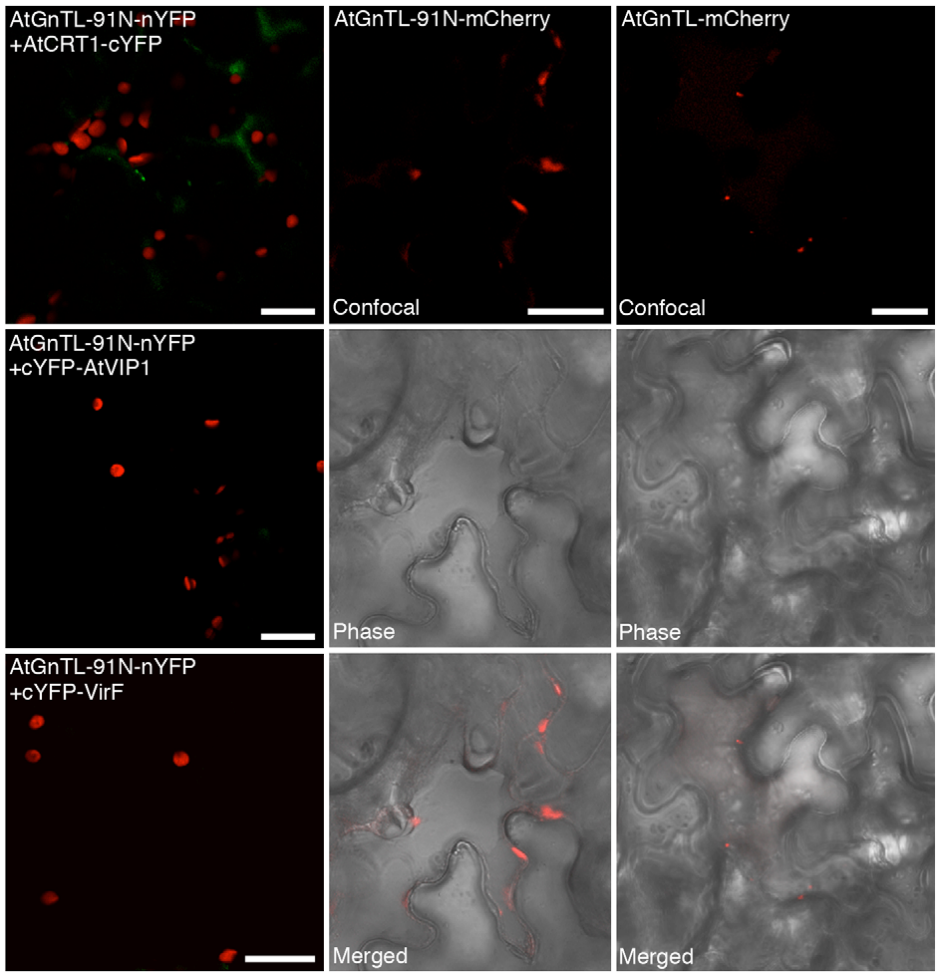
Binding of the amino-terminal domain of AtGnTL to AtCRT1 and its subcellular localization in *N. benthamiana*. Protein interaction was assayed by BiFC. The AtGnTL-91N domain contains amino acid residues between positions 1 and 91 of the AtGnTL protein. YFP fluorescence is in green, plastid autofluorescence is in red. In AtGnTL-91N-mCherry and AtGnTL-mCherry subcellular localization experiments, mCherry fluorescence is in red, and plastid autofluorescence was filtered out. Images are single confocal sections. Bars = 20 µm.

Next, we partially uncoupled between the AtGnTL-AtCRT1 binding and Pd localization. Specifically, we identified a domain within AtGnTL (AtGnTL1-91N), which comprises 91 amino-terminal residues of this protein, that still interacted with AtCRT1 in the BiFC assay in living plant cells (Fig. 6). The interaction between the nYFP-tagged AtGnTL1-91N mutant and AtCRT1-cYFP was specific because it was not observed with unrelated proteins, cYFP-VIP1 and cYFP-VirF (Fig. 6). Fig. 6 also shows that the AtGnTL1-91N-nYFP-AtCRT1-cYFP complexes accumulated at the cell periphery in a somewhat more diffused fashion than the AtGnTL1-91N-nYFP-AtCRT1-cYFP complexes (see Fig. 2B). These observations suggest that this amino-terminal domain of AtGnTL retains its ability to bind AtCRT1, but is at least partially compromised in its ability to traffic to and/or accumulate at Pd. The defect in Pd localization of AtGnTL1-91N was even more dramatic when this protein domain, tagged with mCherry, was expressed alone. Fig. 6 shows that AtGnTL1-91N-mCherry localized to large patches at the cell periphery, never forming the Pd-type puncta, which were clearly seen in parallel experiments with AtGnTL1-mCherry. Collectively, the data in Fig. 6 suggest that the AtGnTL1-91N has lost most of the ability of the full length AtGnTL to target to Pd, and that its interaction with AtCRT1 partially restored Pd targeting, potentially, by using the Pd localization activity of AtCRT1.

### AtGnTL affects plant growth and is expressed in the areal parts of the plant

**Figure 7:**
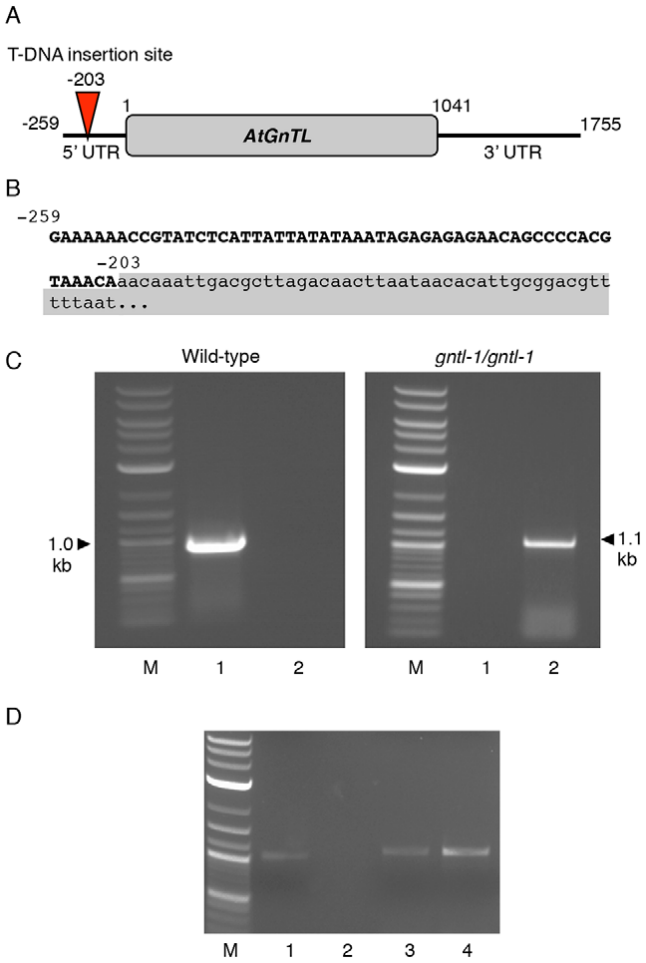
Characterization of the *gntl-1 Arabidopsis* mutant line. (A) Location of the mutagenic T-DNA insertion in the 5′ UTR region of the *AtGNTL* gene. Positions of nucleotide residues delimiting the gene and its UTR regions are shown. (B) Mutagenic T-DNA insertion in the *gntl-1* line. *AtGNTL*-T-DNA right border integration junction sequence is shown, in which the T-DNA sequence is shown in lower case and shaded; the nucleotide positions of the *AtGNTL* sequence are indicated. (C) PCR analysis of the wild-type and homozygous *gntl-1* plants. Lanes M, molecular size markers; lanes 1, *AtGNTL* UTR-specific primers; lanes 2, *AtGNTL* UTR/T-DNA-specific primers. The wild-type *AtGNTL* gene and its *gntl-1* allele containing the mutagenic T-DNA are represented by 1.0-kb and 1.1-kb PCR products, respectively. (D) RT-PCR analysis of the wild-type and homozygous *gntl-1* plants. Constitutively expressed *TUBULIN* gene was used as internal control. Lane M, molecular size markers; lanes 1, 2, wild-type and homozygous *gntl-1* plants, respectively, analyzed with *AtGNTL* mRNA-specific primers; lanes 3, 4, wild-type and homozygous *gntl-1* plants, respectively, analyzed with *TUBULIN* mRNA-specific primers.

**Figure 8:**
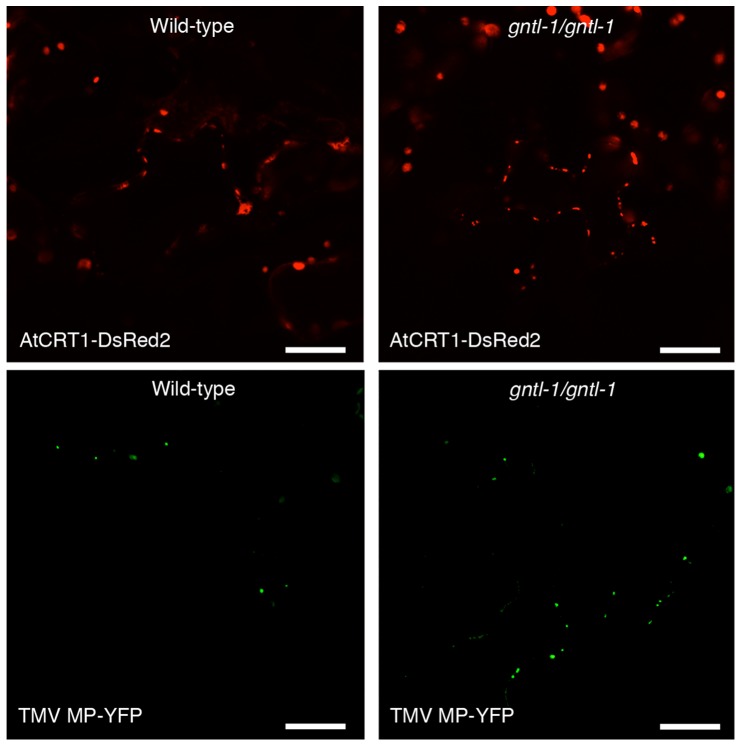
Pd localization of TMV MP-YFP and AtCRT1-DsRed2 in the wild-type and homozygous *gntl-1 Arabidopsis* plants. YFP fluorescence is in green, DsRed2 fluorescence is in red; plastid autofluorescence was filtered out. Images are single confocal sections. Bars = 20 µm.

To better understand the biological role of AtGnTL, we isolated an Arabidopsis mutant, designated *gntl-1*, from the Salk collection [43] with a T-DNA insertion in the 5′UTR sequence of the *AtGnTL* gene (Fig. 7A, B). We then produced homozygous *gntl-1* plants (Fig. 7C) and used RT-PCR to demonstrate that, unlike the wild-type plants, this *gntl-1* line did not express the *AtGnTL* mRNA whereas both wild-type and mutant plants produced transcripts specific for a constitutively expressed *TUBULIN* gene (Fig. 7D). Interestingly, the absence of *AtGnTL* expression in the *gntl-1* mutant did not detectibly alter the Pd-specific localization pattern of TMV MP or AtCRT1 (Fig. 8), indicating that AtGnTL is not required for transport of these proteins to Pd. Thus, that AtGnTL1-91N prevented Pd targeting of AtCRT1 (see Fig. 6) is most likely due to steric interference between these two proteins.

**Figure 9:**
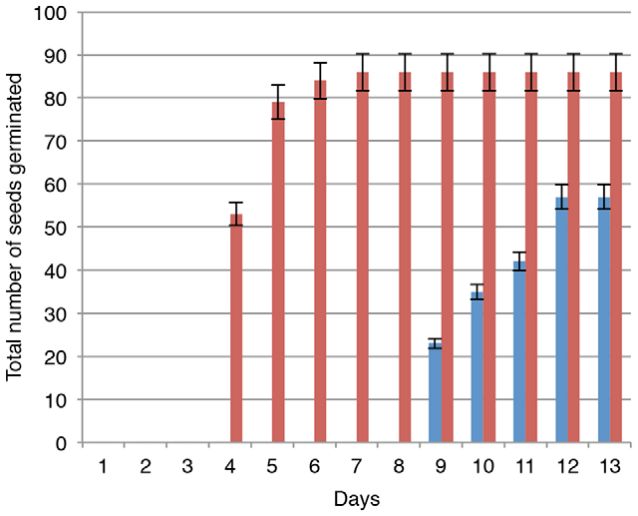
Loss of AtGNTL function leads to reduced seed germination frequency. Red and blue bars represent the wild-type and homozygous *gntl-1 Arabidopsis* plants, respectively. Both types of seeds were collected simultaneously from the parental plants grown under identical conditions. The data were represent average values of three independent experiments with indicated standard deviations.

**Figure 10:**
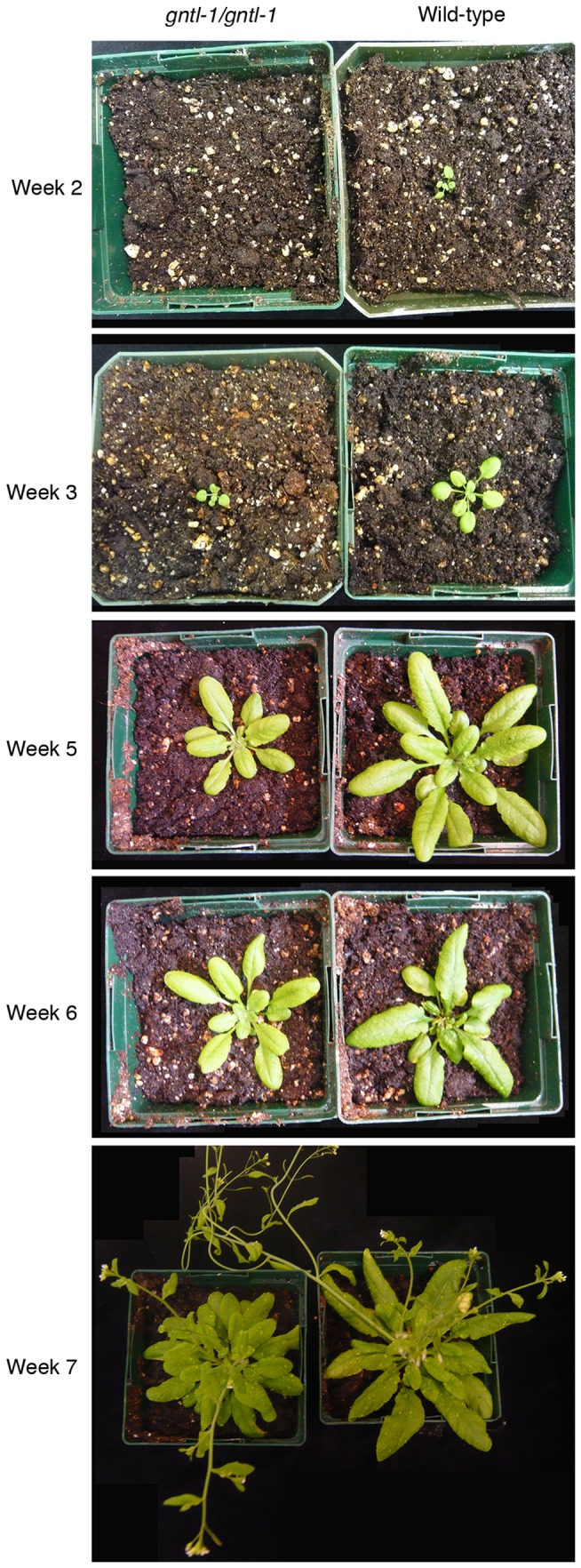
Loss of AtGNTL function leads to delayed plant growth. Representative images of the wild-type and homozygous *gntl-1 Arabidopsis* plants are shown at the indicated times post germination.

The major apparent phenotypic characteristic of the *gntl-1* mutant was its reduced seed germination frequency and delayed plant growth (Fig. 9). Specifically, when seeds of the wild-type and *gntl-1* mutant plants were plated on agar media and germination frequencies of each population were measured, nearly 90% of the wild-type seeds germinated within six days, whereas only up to 60% of the *gntl-1* mutant seeds germinated even after 13 days (Fig. 9). Once germinated, the mutant seedlings exhibited significantly delayed growth until week 5 post germination. At that time, the mutant plants approached the size of their wild-type counterparts, albeit never reaching it completely (Fig. 10). At present, we do not have sufficiently detailed knowledge of the AtGnTL function in the plant to explain the mutant phenotype. Obviously it is likely related to potential defects in glycan synthesis either on the global plant scale or in specific tissues; for example, mutations in a different type of glucosyltransferase has been shown to cause defective seed phenotypes [44].

**Figure 11:**
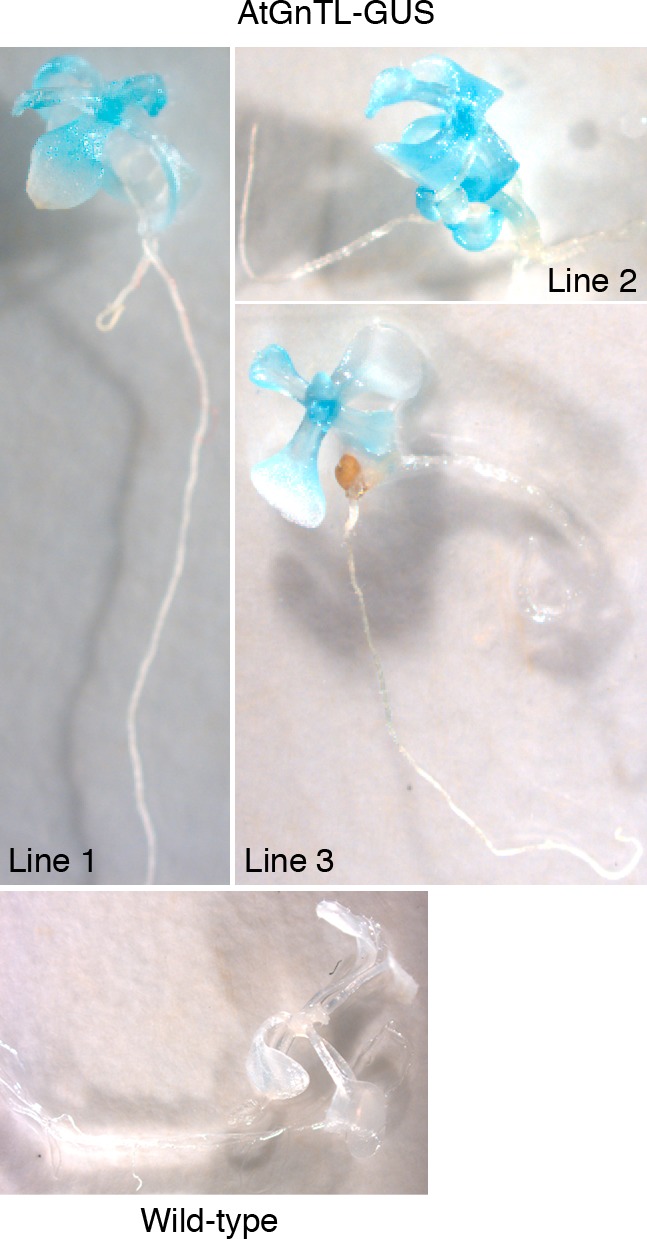
Expression of the *GUS* reporter from the *AtGnTL* promoter in Arabidopsis transgenic plants. Line numbers indicate independent transgenic transformants.

Finally, we analyzed the overall expression pattern of *AtGnTL* in Arabidopsis plantlets. To this end we produced three independent lines of Arabidopsis plants transgenic for AtGnTL tagged with the ß-glucuronidase (GUS) reporter and driven by the *AtGnTL* native regulatory sequences. Fig. 11 shows that all three lines exhibited relatively strong *GUS* expression throughout the shoot, but not in the root areas. As expected no GUS activity was detected in control, wild-type plantlets (Fig. 11). Thus, the native *AtGnTL* gene expression is most likely restricted to the areal parts of the plant. The biological rationale for this expression pattern remains to be elucidated.

## Discussion

The important role of Pd in many aspects of the plant life cycle combined with our relatively limited understanding of the mechanisms of Pd function call for identification of their full protein complement. One way to approach this task is by employing an already known Pd protein as a lead to isolate other, otherwise unpredictable, proteins that attach to it and, thus, are expected also to associate with Pd. Using this rationale, we identified an interactor of AtCRT1, an ER chaperone found in Pd [15]–[18]. Subcellular localization experiments showed that this protein, AtGnTL, an annotated member of the glycosyltransferase superfamily, indeed represents a novel type of Pd-associated enzymes. Beta-1,6-N-acetylglucosaminyl transferases play crucial roles in glycan synthesis [45] and are known to catalyze attachment of oligosaccharide side chains to glycoproteins [46]. That a beta-1,6-N-acetylglucosaminyl transferase is involved in Pd function makes biological sense. Increasing evidence indicates that sugar-containing molecules, such as glucans and glycans, take part in Pd biogenesis and regulation. For example, among other factors, Pd permeability is modulated by callose (a beta-1,3-glucan) sphincters surrounding the Pd orifices [40], [47]–[50], and glycosylation has been implicated in control of protein transport through Pd [51], [52]. Although the specific role of AtGnTL in Pd function requires further experimentation, it is tempting to speculate that this putative enzyme may act to modify other Pd component(s) or even the transported proteins en route to the neighboring cells. The latter possibility is especially intriguing as it would suggest that Pd not only transport proteins between cells, but also participate in post translational modification of the transport substrates such that a molecule that enters the “recipient” cell is biochemically different from the same molecule that has left the “donor” cell. This cargo modification most likely would occur within trans-Pd ER, a strand of ER that traverses Pd [4] and in which AtGnTL and its interactor AtCRT1 most likely are located.

The ability of AtGnTL to interact with AtCRT1 suggests that AtCRT1 may play a role in the AtGnTL function at Pd, for example by acting as a chaperone, a known activity of calreticulin [53], [54], in presenting the AtGnTL substrates. Indeed, both proteins likely reside in the ER, with AtCRT1 being a known ER chaperone [53], [54] and AtGnTL containing a signal peptide (see Fig. 1B, C), one of the hallmarks of ER proteins [28], [29]. Within the ER they may cooperate, potentially along with calnexin and other chaperones known to function together with glucosyltransferases, in glycoprotein folding and quality control [55]. Alternatively, AtCRT1 may simply chaperone the AtGnTL protein itself, helping it to fold properly.

Regardless of the exact mechanism of the AtGnTL action, the observation that a putative glycosyltransferase-like protein may reside and function at or around Pd suggests that plant intercellular connections are not just conduits for macromolecular transport but also represent sites for glycan synthesis and, potentially, post translational modification.

## Materials and Methods

### Plants

Wild type *A. thaliana* (Columbia-0 ecotype) and the *gntl-1* T-DNA insertion line (SALK_012392.54.50.X, obtained from ABRC), and *N. benthamiana* plants were used in the experiments. All plants were grown under the same conditions in soil in an environment-controlled chamber at 22–24°C under long day conditions of 16 h white light (70–80 µmol photons m-2 s-1) and 8 h dark. At least 10 plants were used for each experiment condition, and all experiments were repeated three times. For PCR-based analyses, *Arabidopsis* genomic DNA was extracted using DNeasy Plant Kit (Qiagen). Then, to identify plants carrying the wild-type *AtGnTL*, we utilized the gene-specific forward and reverse primers 5′-AGGGGAATAATGACGTCAGCAAAA-3′ and 5′-AGCTGAGATGTTGCCAGGAGAAGT-3′, respectively, whereas plants homozygous for T-DNA insertion in this gene, the *gntl-1* mutant, were identified using the gene-specific forward primer 5′-AGGGGAATAATGACGTCAGCAAAA-3′ and the T-DNA left border-proximal reverse primer 5′-ATCAAACAGGATTTTCGCCTGCT-3′.

### Y2H assay

For bait construct, a *AtCRT1* sequence (GenBank accession number NM_104513.2) fragment containing amino acid residues between positions 21 to 38, a *CFP* linker, and residues 415 to 425 was cloned into the BglII-SalI sites of a LexA plasmid pSTT91 (TRP1+) [56]. *Arabidopsis* cDNA library in pGAD424 (LEU2+, Clontech), as well as human lamin C and topoisomerase I in pSTT91 were described previously [26], [27], [57]. The *AtCRT1* cDNA was cloned into the SmaI-PstI sites of pSTT91, and the *AtGnTL* cDNA was cloned into the EcoRI-SalI sites of pGAD424; to allow efficient Y2H transcriptional activation, both cDNA clones lacked their 25-bp and 30-bp 5′-terminal sequences, respectively, coding for the amino-terminal signal peptides. All plasmids were introduced into yeast cells using a standard lithium acetate protocol [58]. Protein interactions were selected in *Saccharomyces cerevisiae* strain L40 (MATa *his3Δ200 trp1-901 leu2-3,112 ade2 lys2-801amas URA3::(lexAop)_4_-HIS3*) [59] by growing cells for 3 days at 30°C on a leucine-, tryptophan- and histidine-deficient medium. All positive interactions were confirmed by a β-galactosidase assay as described [60].

### Agroinfiltration and microbombardment

For agroinfiltration of *N. benthamiana*, binary plasmids were introduced into the Agrobacterium strain GV3101 [61] grown overnight at 25°C and infiltrated into intact leaves as described [62], [63]. For biolistic delivery of *Arabidopsis*, 100 µg DNA was absorbed onto 10 mg of 1-µm gold particles (Bio-Rad) and microbombarded into leaf epidermis at a pressure of 90–150 psi using a portable Helios gene gun system (Model PDS-1000/He, Bio-Rad) essentially as described [41]. After incubation for 36–48 h at 22–24°C, the agroinfiltrated or microbombarded tissues were viewed under a Zeiss LSM 5 Pascal confocal laser scanning microscope. All PCR reactions were performed using a high-fidelity *Pfu* DNA polymerase (Stratagene) and products were verified by DNA sequencing. All experiments were repeated at least three times.

### Confocal microscopy

Specimen preparation and imaging were performed as described [39]. Briefly, plant tissue samples were mounted in water between number 1 1/2 coverglasses, using silicon vacuum grease to create spacers between the glass surfaces. Images were collected with a Zeiss LSM 5 Pascal laser scanning confocal microscope system. In all cases, a high numerical aperture (1.2–1.3) water immersion objective (60–63x) was employed. A 458 nm, 488 nm, or 514 nm line from an argon ion laser was used to excite CFP, GFP, and YFP, respectively, and 543 nm or 587 nm line from an helium-neon ion laser was used to excite DsRed2 or mCherry, respectively. All image acquisition, i.e., laser intensity and photomultiplier tube (PMT), settings were preserved between different experiments. On average 100–120 cells were analyzed for each experiment.

### BiFC

The full-length *AtCRT1* cDNA was cloned into the BglII-SalI sites of pSAT4A-cEYFP-N1 GenBank accession number DQ169002). The full-length *AtGnTL* cDNA and the *AtGnTL1-91N* sequence coding for the first 91 amino acid residues of AtGnTL were cloned into the XhoI-KpnI sites of pSAT4A-nEYFP-N1 (GenBank accession number DQ169003). Note that, in both of these tagged constructs, the fusion proteins retain their native amino-terminal signal peptide sequences. cYFP-AtVIP1 and cYFP-VirF fusion constructs were described previously [64], [65]. The tested construct pairs were mixed 1∶1 (w/w) and microbombarded into *N. benthamiana* leaves, allowed to express for 36–48 h at 22–24°C, and analyzed by confocal microscopy.

### Protein localization

The full-length *AtGnTL* cDNA was cloned into the XhoI-KpnI sites of pSAT6-EGFP-N1 (GenBank accession number AY818382). The *mCherry* coding sequence (GenBank accession number JQ627840) was cloned into the KpnI-BamHI sites of pSAT5-MCS – which is identical to pSAT6-MCS (GenBank accession number AY818383), except that the entire expression cassette is flanked by I-CeuI [66]– to produce pSAT5-mCherry-N1. The *AtGnTL1-91N* cDNA was then cloned into the XhoI-KpnI sites of pSAT5-mCherry-N1. For native promoter construct, we first replaced the CaMV 35S promoter in pSAT5-MCS with a PCR-amplified 1.0-kb AgeI-XhoI fragment that contained the entire intergenic region of *AtGnTL*. Then, the coding sequences of *AtGnTL* and *mCherry* were cloned into the XhoI-KpnI and KpnI-BamHI sites, respectively, of this vector. For agroinfiltration, the expression cassettes were excised with AgeI or I-CeuI from their pSAT6- or pSAT5-based vectors, respectively, and each inserted into a separate pPZP-RCS2 binary vector [66], [67]. For microbombardment, the proteins were expressed directly from the pSAT-based vectors. The construct expressing TMV MP-YFP was described previously [40]. The *AtCRT1-DsRed2* expression construct based on the pSAT4 plasmid [66] was a generous gift from Dr. Ueki (Okayama University). The tested constructs were agroinfiltrated into *N. benthamiana* or microbombarded into *Arabidopsis*, incubated for 36–48 h at 22–24°C, and observed by confocal microscopy. For quantification, we counted the total number of puncta in confocal images for each of the individual proteins (i.e., AtGnTL-GFP, AtPDCB1-mCherry, and TMV MP-YFP) as well as those puncta that show colocalization on merged images. We then calculated the percentage of the colocalized signal relative to total number of puncta for each protein.

### Plasmolysis

The *AtGnTL* cDNA was inserted into the XhoI-KpnI sites of pSAT5-mCherry-N. *CFP* coding sequence was subcloned from pRSET-CFP (Invitrogen) into the NcoI-XhoI sites of pSAT4-MCS (GenBank accession number DQ005466.1). The resulting *AtGnTL-mCherry* and *CFP* expression cassettes were excised with I-CeuI or I-SceI from their pSAT5- or pSAT4-based vectors, respectively, and each inserted into a separate pPZP-RCS2 binary vector. The resulting binary constructs were mixed at 1∶1 (w/w) ratio and transiently expressed for 36–48 h at 22–24°C in *N. benthamiana* leaves following agroinfiltration. For plasmolysis, leaf sections were excised, incubated in 0.45 M mannitol as described [68] until epidermal cells were visibly plasmolysed, and examined by confocal microscopy.

### Protein colocalization

The *AtGnTL-GFP* expression cassette was excised PI-PspI from pSAT6-EGFP-N1 and inserted into pPZP-RCS2. The *AtPDCB1-mCherry* binary construct [22] was kindly provided by Dr. Maule (John Innes Centre). The tested binary constructs, i.e., those expressing AtGnTL-GFP and AtPDCB1-mCherry or TMV MP-YFP and AtGnTL-mCherry, were mixed at 1∶1 (w/w) ratio and transiently expressed for 36–48 h at 22–24°C in *N. benthamiana* leaves following agroinfiltration, and protein subcellular localization was analyzed by confocal microscopy.

### RT-PCR

For RT-PCR, total RNA from two-week old seedlings was isolated with TRI-reagent (Molecular Research Center), treated with RNase-free DNase (DNA-free kit, Ambion), and 0.5 µg of purified DNA-free RNA was reverse-transcribed with ProtoScript First Strand cDNA synthesis kit (New England Biolabs) and PCR-amplified for 28–32 cycles using *AtGnTL* 5′UTR- specific forward and reverse primers 5′-ATGTTCTCATCTTCTACACTCGTTTATTC-3′ and 5′-TCAATCCCTAAAGATCACAGCATCTGC-3′, respectively. The absence of contaminating genomic DNA was confirmed by PCR using *TUBULIN*-specific forward and reverse primers 5′-AGATTCTTCACATCCAGGGTGGTC-3′and 5′-CTCACTCACTCGCCTGAACATCTC-3′, respectively, that flank an intron sequence to distinguish between PCR products derived from DNA and mRNA templates [69]; *TUBULIN* also served as an internal control of a constitutively expressed gene. These RT-PCR reactions amplified 1,041-bp and 1,141-bp products from the *AtGnTL* and *TUBULIN* transcripts respectively.

### Generation of transgenic Arabidopsis plants and GUS activity assay

For production of plants that express the AtGnTL-GUS fusion protein from the native *AtGnTL* regulatory elements, we utilized the 1-kb *AtGnTL* sequence upstream of the ATG codon, based on the size of the predicted *AtGnTL* intergenic region. This region was amplified from the wild-type *Arabidopsis* genomic DNA with the forward and reverse primers 5-ACCGGTAGCTGAGATGTTGCCAGGAG-3′ and 5′-CCGCTCGAGCTCTCTGTATACAACAACAC-3′ and cloned into the AgeI-KpnI sites of the pSAT4-35SP-MCS-35ST-GUS vector, replacing the 35S promoter; pSAT4-35SP-MCS-35ST-GUS is based on the pSAT4-35SP-MCS-35ST vector [70] and was kindly provided by Dr. Adi Zaltsman (Stony Brook University). Next, the cDNA sequence of AtGnTL with its own translation initiation codon was inserted into the XhoI-KpnI sites downstream of the *AtGnTL* native promoter and in-frame to the *GUS* coding sequence. The resulting expression cassette was then transferred into the I-SceI site of the pPZP-RCS2 binary vector [66], [67], containing the *bar* gene for BASTA selection in its XhoI-BamHI sites [71]. This binary construct was introduced into the Agrobacterium EHA105 strain, used to transform the wild-type Arabidopsis plants by flower dipping [72], and transformants were obtained using BASTA selection.

To visualize GUS activity, transgenic Arabidopsis seedlings at the 4-leaf stage aseptically grown in baby food jars in MS agar with 5 mg/l BASTA [73] were assayed histochemically as described [73], and recorded under a Leica MZ FLIII stereoscope.
